# ‘An extra level of kind of torment’: Views and experiences of recurrent miscarriage care during the initial phases of COVID‐19 in Ireland—A qualitative interview study

**DOI:** 10.1111/hex.13791

**Published:** 2023-06-09

**Authors:** Rebecca Dennehy, Marita Hennessy, Jennifer Ui Dhubhgain, Con Lucey, Keelin O′Donoghue

**Affiliations:** ^1^ Pregnancy Loss Research Group, Department of Obstetrics and Gynaecology University College Cork Cork Ireland; ^2^ INFANT Research Centre University College Cork Cork Ireland; ^3^ RE:CURRENT Research Advisory Group, Pregnancy Loss Research Group, Department of Obstetrics and Gynaecology University College Cork Cork Ireland; ^4^ Miscarriage Association of Ireland Carmichael Centre Dublin Ireland

**Keywords:** COVID‐19, maternity care, miscarriage, pregnancy loss, qualitative, recurrent miscarriage

## Abstract

**Introduction:**

Maternity services underwent much change during the COVID‐19 pandemic. Research on the impact on miscarriage care and experiences during this time is sparse. Within a national evaluation of recurrent miscarriage care, we qualitatively explored stakeholder views and experiences of recurrent miscarriage services in Ireland. This study describes the impact of the COVID‐19 pandemic on those experiences and perceptions of care.

**Methods:**

People with professional and lived experience of recurrent miscarriage and service engagement were actively involved in this qualitative study from idea generation to analysis and reporting. We recruited women and men with two or more consecutive first‐trimester miscarriages, and people involved in the management/delivery of recurrent miscarriage services and supports. We used purposive sampling to ensure that perspectives across disciplinary or lived experience, geographical, and health service administrative areas, were included. We conducted semi‐structured interviews, virtually all due to COVID‐19 restrictions, between June 2020 and February 2021. These were audio‐recorded, and data were transcribed, and subsequently analyzed using reflexive thematic analysis.

**Results:**

We interviewed 42 service providers and 13 women and 7 men with experience of recurrent miscarriage. We actively generated two central themes during data analysis. The first—‘Disconnected’—describes how many women navigated miscarriage diagnosis and management and care in subsequent pregnancies alone; many felt that this resulted in increased trauma. At the same time, men struggled with not being present to support their partners and described feeling disconnected. The second theme highlighted ‘The perceived dispensability of recurrent miscarriage services and supports’. Some service providers felt that service reduction and redeployment demonstrated a lack of value in the service. Virtual clinics facilitated access to services, but a preference for in‐person care was highlighted.

**Conclusion:**

Our analysis provides rich insights into the significant impacts that the COVID‐19 pandemic has had on the way recurrent miscarriage care is provided and experienced, with important implications for early pregnancy, miscarriage and recurrent miscarriage care. Services have undergone significant changes and, while these may be temporary, how services should be delivered in the future requires consideration, particularly given the deficits in care and care experiences highlighted prepandemic.

**Patient or Public Contribution:**

Members of the multidisciplinary RE:CURRENT Project Research Advisory Group (including four parent advocates, two of whom are co‐authors on this article) were actively involved throughout the study, including the generation of topic guides and the refining of themes.

## 1 INTRODUCTION

The global pandemic caused by severe acute respiratory syndrome coronavirus 2 (SARS‐Cov‐2), or ‘COVID‐19’ has, and indeed continues to have, far‐reaching health, social and economic impacts. Across the world, maternal and foetal outcomes have been negatively impacted, with increased rates of maternal deaths, stillbirth, ruptured ectopic pregnancies and maternal depression observed.^1^ Reduced maternity healthcare‐seeking and healthcare provision has also been noted globally, and could be a potential contributory factor in these outcomes.^2^ The COVID‐19 pandemic resulted in changes in how health services were delivered and experienced due to efforts to restrict COVID‐19 transmission and manage constrained healthcare resources. Many changes have taken place within maternity and perinatal bereavement care services due to COVID‐19.^3^, ^4^ Guidance issued within countries and by major international bodies was the subject of ongoing, rapid change and there were differences in the recommendations, adding to confusion.^4^ Though some positive changes were evidenced during COVID‐19,^5^, ^6^ changes within maternity care were negatively experienced by women and maternity care providers.^6^

Impacts on maternity care and pregnancy, birth and/or postpartum experiences during the pandemic have been documented in several countries or regions internationally,^6^ including Ireland,^7^, ^8^, ^9^, ^10^ the United States,^5^, ^11^ United Kingdom,^12^, ^13^, ^14^, ^15^, ^16^ Australia,^17^, ^18^ the Netherlands^19^, ^20^ and Europe.^21^ Changes within maternity care common to many countries, and reported areas of concern, included transition to virtual appointments or consultations for prenatal care and/or reduced appointments, and restrictions on visitors, partners or support persons attending prenatal scans and appointments or procedures.^5^, ^7^, ^11^, ^12^, ^13^, ^14^, ^16^, ^17^, ^18^, ^19^, ^20^, ^21^, ^22^ In their study, McKinlay et al.^13^ noted that some participants felt that they did not receive the same quality of maternity service care during the pandemic compared to prepandemic times. Participants in a US study expressed concerns about the lack of quality of maternal care, whether feeling like they were not prioritised in the medical system due to preventing/treating COVID‐19 or because of a shift to telehealth appointments.^11^ While studies show that women find some benefits to telehealth, overall it is viewed as problematic and less favoured than in‐person care, with concerns raised about the potential for important information being missed, particularly by women who have pregnancy‐related issues, including a previous stillbirth or miscarriage.^6^ Connection, including trust, touch and support from staff, was perceived by pregnant women using UK maternity services to influence care experiences and satisfaction.^13^ In their study, McKinlay et al.^13^ noted that some people were more likely to be upset about partners being excluded from healthcare appointments and/or interactions; these included people experiencing miscarriage, and people with previous miscarriage. There is limited evidence to‐date on how fathers or support partners experienced maternity care during the pandemic.^17^

Negative impacts of restrictions on maternity services and how they were experienced have also been the subject of advocacy efforts, led by academics, clinicians, people with lived experience and advocacy groups. Many have argued for more compassionate, person‐centred policies and approaches within maternity services,^7^, ^23^ underpinned by human rights,^4^ rather than basing decisions on risk assessments that do not factor in broader impacts. The need for guidelines in maternity care regarding COVID‐19 to consider women experiencing pregnancy after loss was also highlighted by Pollock et al.^22^ Throughout the pandemic, restrictions continued in many maternity services, despite the lack of evidence to justify these^4^, ^16^ with increased levels of vaccination against COVID‐19, and a better understanding of the virus in general.

The need to support health care workers and maternity care providers to continue to provide the best quality care while adapting to a rapidly changing health system environment, and indeed throughout the pandemic, has also been highlighted.^24^ A global survey of maternal and newborn health professionals conducted during March–April 2020 found increased levels of stress among health professionals due to various factors including changed working hours, and staff shortages.^25^ Healthcare providers expressed concern about the impact of rapidly changing care practices on health outcomes, including reduced access to antenatal care, fewer outpatient visits, shorter length of stay after birth and partner restrictions.^25^ While staff could experience moral injury due to the nature of some of the restrictions imposed; for some, this may have coexisted alongside feelings of relief that they were personally being protected from COVID‐19 infection by these same restrictions.^26^ In Australia, Bradfield et al.^17^ observed differences in the experiences of those who received, versus those who provided, care during the pandemic—with the former being much more concerned about the changes to maternity care.

Increased social isolation, limited social and practical support and negative mental health effects arising from the pandemic, in part due to changes to medical care perceived as abrupt or random, have been noted.^5^, ^12^, ^13^ In an Irish study, women who were pregnant during the COVID‐19 pandemic reported lower perceived social support (including support from a significant other, friends and family), than women pregnant before the pandemic.^27^ People who experience a pregnancy loss or perinatal death require particular care and support. There are many and varied psychological impacts associated with different types of pregnancy loss, including miscarriage and stillbirth.^28^, ^29^ People who had experienced miscarriage were featured in a study by McKinlay et al.^13^ on the experiences of pregnant women using UK maternity services during the pandemic, in which the impact of social distancing rules on mental health and wellbeing was highlighted. Some studies specifically documenting experiences of, and/or impacts on, people who experienced a late miscarriage, stillbirth or neonatal death during COVID‐19 have been undertaken, with some findings published^3^, ^30^ and some forthcoming.^31^, ^32^ Efforts to minimise impacts on care quality included virtual consultations, online staff training, use of cold cots and increased staff support for memory‐making.^3^ A qualitative study conducted with bereavement midwives in Ireland highlighted unique challenges when providing perinatal bereavement care; mandatory COVID‐19 measures significantly disrupted human communication and connections (facemasks, social distancing and lack of personal touch) with staff doing their best to provide optimum care, while at the same time dealing with their own fears and anxieties.^8^ Preliminary findings from the PUDDLES study on the experiences of care for parents who experienced a late miscarriage, stillbirth or neonatal death during the COVID‐19 pandemic demonstrate that maternity, neonatal and bereavement care services were reconfigured and access reduced, with parents' experiences particularly affected.^30^ Thus, much literature exists on the impacts of COVID‐19 on maternity care experiences, and evidence is building regarding later pregnancy loss. There remains, however, limited empirical research documenting the experiences of miscarriage care during the COVID‐19 pandemic despite media attention and advocacy efforts focused on this area across many countries.

In this study, we present analysis of data gathered during June 2020 and February 2021 on the views and experiences of people with personal or professional experience of recurrent first‐trimester miscarriage, focusing specifically on the impact of COVID‐19 on services and supports.

## 2 METHODS

Our analysis draws on data from a large qualitative interview study, conducted with people involved in the management and/or delivery of services and support (within hospital and community or voluntary settings) and people with lived experience of recurrent miscarriage, to explore their views and experiences of recurrent miscarriage services and support. We adopted social constructionism as the theoretical underpinning for this study, acknowledging that knowledge and meaning are socially produced.^33^ The study methods are outlined briefly below. A separate analysis of how participants think recurrent miscarriage is and/or should be defined has been published,^34^ along with a paper outlining ethical considerations regarding outsourcing data transcription.^35^ A further paper documenting stakeholder perspectives on recurrent miscarriage services and priorities for improvement is forthcoming.^36^ This qualitative study formed part of a larger evaluation of recurrent miscarriage services in the Republic of Ireland (the RE:CURRENT Project).

We undertook this analysis of views and experiences of services and supports during COVID‐19 separately, as it was an important feature of the broader RE:CURRENT qualitative study, and merited an exploration and illumination, in its own right. Participants were not directly questioned about COVID‐19; however, the study took place during the pandemic and participants often alluded to it during discussions.

### 2.1 Study context

Data were collected between June 2020 and February 2021, during the first year of the COVID‐19 pandemic where there were varying and significant levels of COVID‐related public health and healthcare restrictions, from initial national lockdown measures to varying national/local restrictions. On 29 February 2020, the first case of the coronavirus was confirmed in the Republic of Ireland and the first death by coronavirus on 11 March, the same day as the World Health Organisation declared the COVID‐19 outbreak a global pandemic.^37^ In March 2020, the Irish Government announced restrictions to manage the COVID‐19 pandemic, including a visiting ban in all hospitals and various travel limitations. In June, when our study commenced, restrictions had begun to be eased further to the publication of the Government's ‘Roadmap for Reopening Society and Business’ on 01 May 2020.^38^ There was a steep decrease in disease incidence from late April, followed by a period of low incidence and relative stability during June and early July, but increasing levels again from July to October 2020^37^ followed by another state‐wide lockdown lasting 6 weeks though schools and childcare facilities remained open.^39^ In early December, restrictions were eased again,^40^ only to be reinforced later that month due to a steep rise in case numbers.^41^ In February 2021, hospital numbers were still at peak levels of wave 2 and the national vaccination programme was gaining momentum, following its introduction in December.^42^

Maternity care services were issued with numerous iterations of guidance on how to best structure and deliver services to keep women, babies, families and staff safe. Guidance issued by the Royal College of Physicians of Ireland regarding maternity care during COVID‐19,^43^, ^44^ on 05 May 2020 and 01 May 2021, reinforced the importance of ensuring that those at risk of early pregnancy complications continued to be seen in the event of any rationalising of ultrasound visits; women should be facilitated to have a partner attend early pregnancy scans with them, especially if they had a previous pregnancy loss or threatened miscarriage; support for women and families, and ensuring staff wellbeing. Guidelines also stated that visiting policies for maternity units should be decided by management in each hospital, following overall Health Service Executive guidance, taking the clinical situation in each individual hospital at the time into account, in particular the presence of local outbreaks and high community spread. The Health Service Executive applied mandatory directives in all hospitals under their remit, which included: social distancing, timing of interactions limited to <15 min, wearing personal protective equipment, visiting restrictions and partners were only permitted to attend the birth of the baby or on compassionate grounds. The Health Service Executive also issued guidance on visitation to acute hospitals; for example, on 31 May 2021, it made explicit reference to miscarriage, stillbirth and other adverse pregnancy outcomes as critical and compassionate circumstances whereby visiting arrangements should be as flexible as possible, cognisant of other safety considerations.^45^ The Health Protection Surveillance Centre issued separate guidance on visitor restrictions for all hospitals under the remit of the Health Service Executive; they advocated local risk assessments and responses which were both pragmatic and proportionate.^46^ In Ireland, the Association for Improvements in Maternity Services (AIMS)‐Ireland advocated around maternity restrictions throughout the pandemic, particularly during the first 2 years given restrictions placed on services, particularly regarding partners.^47^ There were also a series of national protests.^48^, ^49^

### 2.2 Reflexivity

The research team comprised a woman (J. U. D.) and a man (C. L.) with lived experience of recurrent miscarriage; and a social scientist (R. D.), a public health/health services researcher (M. H.), and an obstetrician and maternal–foetal medicine subspecialist (K. O. D.), all with experience in conducting pregnancy loss and/or maternal and child health research. Both J. U. D. and K. O. D. were involved in the provision of services and/or supports to people with recurrent miscarriages throughout the COVID‐19 pandemic; J. U. D. as part of her voluntary role with the Miscarriage Association of Ireland, and K. O. D. clinically in her role as an obstetrician and clinical lead of pregnancy loss services (including a dedicated recurrent miscarriage clinic) at Cork University Maternity Hospital. K. O. D. was also the lead author on COVID‐19 guidelines for maternity services during the pandemic.^43^ Both C. L. and R. D. had lived experience of maternity service use during the COVID‐19 pandemic. All of the team were acutely aware of the impact of COVID‐19 restrictions on service provision and care experiences, and the need to better support people in this regard, particularly people who were experiencing pregnancy loss or pregnancy after loss. We brought these beliefs to the study and engaged fully with the data to ensure that our pre‐existing beliefs did not unduly influence our analysis.

### 2.3 Participants

We purposively sampled people involved in the delivery/management of recurrent miscarriage services and supports, and women and men/partners with lived experience of at least two consecutive first‐trimester miscarriages (most recent, within 6–24 months before study participation), in the Republic of Ireland. We wanted to ensure maximum variation and sampled from different geographical areas and settings. In the Republic of Ireland, recurrent miscarriage is generally defined as three consecutive miscarriages, but there is variation in how this is applied in practice around the country, and how services are structured also;^50^ we wanted to capture this in our sample. We included biochemical pregnancies as well as those documented by ultrasonography or histopathological examination in our definition. We recruited professionals via our networks, including project collaborators, while people with lived experience of recurrent miscarriage were recruited via health professionals and social media.

### 2.4 Data collection

We (R. D. and M. H.) conducted semi‐structured interviews with participants via Zoom. Two topic guides were used: one specific to women and men with lived experience, covering a range of topics including experiences and impacts of recurrent miscarriage (including their first and subsequent miscarriages), investigations and treatments and care in subsequent pregnancies.^34^ We audio‐recorded interviews and took detailed field notes following each interview. Audio files were professionally transcribed verbatim. After checking and removing any identifying details and assigning pseudoanonyms, they were imported into NVivo 12 for data management and analysis. We ceased sampling when the major categories showed depth and variation—both within and across interviews both participant groupings, that is, when we felt we had achieved ‘information power’.^51^

### 2.5 Data analysis

We used reflexive thematic analysis to develop patterns of meaning (‘themes’) across the data set through a rigorous process of data familiarisation, through reading and rereading the transcripts; data coding, inductive; generating initial themes; theme development and revision; and writing up.^33^ The analysis was led by R. D., but was conducted using a team‐based approach with other members of the research team who met frequently to discuss the analysis during each phase. We conducted the analysis of data from both participant groupings (people with lived experience and those involved in the delivery/management of services and supports) together as similar issues were arising within their accounts, providing multifaceted insights. Coding of data relating to the research question relating to views and experiences of services and supports during COVID‐19 was conducted at the same time as the main analysis. From there, the analysis diverged into separate studies and is reported separately.^34^

## 3 RESULTS

We included data from interviews with 42 health professionals/service providers and 13 women and 7 men with lived experienced recurrent miscarriage in our analysis. Health professionals/service providers encompassed: Clinical Midwife/Nurse Specialists in Bereavement and Loss (CMS; *n* = 8); Consultant Obstetricians/Gynaecologists (hospital‐based) (OBGYN‐H; *n* = 4); Consultant Obstetrician (private fertility sector) (OBGYN‐FC; *n* = 1); Specialist Registrars (*n* = 2); Nurses (GN), Midwives (M), Sonographers (S) (*n* = 4); Chaplaincy and Pastoral Care (CP; *n* = 2); Support Services (Perinatal Mental Health [PMH], Social Work, Community and Voluntary [VS] [*n* = 3]); GPs (GP; *n* = 4); Practice Nurses (*n* = 2); Public Health Nurses (*n* = 2); Administrative Support (*n* = 1); Maternity Hospital/Unit/Group Level Administration, Governance and Management—Directors of Midwifery; Group Director of Midwifery (*n* = 3); National Administration, Governance and Management (AGM) (*n* = 6). The 20 women and men we interviewed had experienced a range of first‐trimester miscarriages (2–15) including numbers of consecutive miscarriages. Fourteen had living children and three were pregnant at the time of the interview.

**Figure 1:**
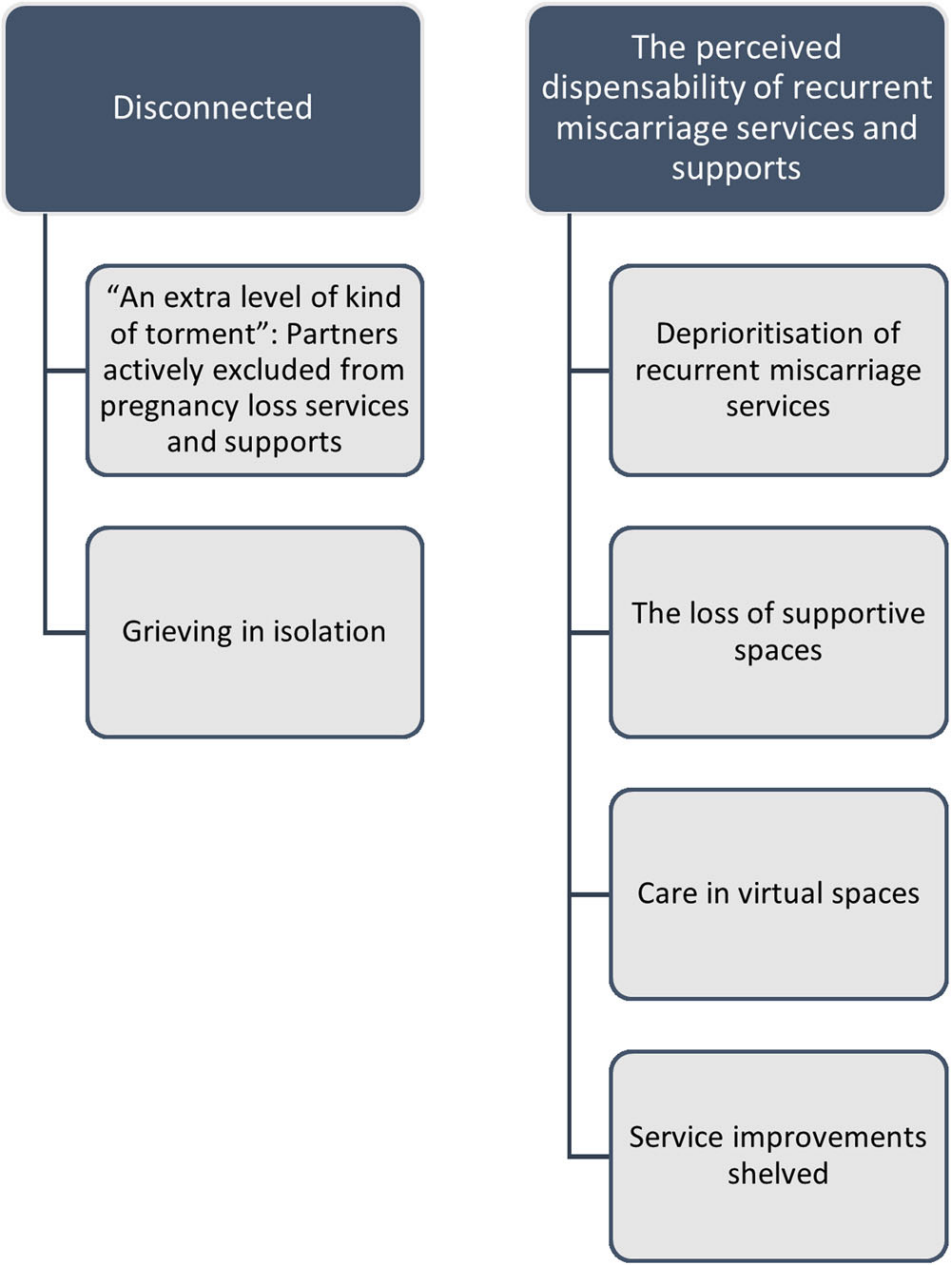
Overview of themes and subthemes.

We generated two central themes regarding people's views and experiences of recurrent miscarriage services and supports during the first 15 months of the COVID‐19 pandemic: ‘Disconnected’ and ‘The perceived dispensability of recurrent miscarriage services and supports’ (Figure 1).

These themes, each with subthemes and illustrative quotes, are outlined in Tables 1 and 2.

**Table 1:** Theme 1: Disconnected.

Subtheme	Illustrative quote	Quote no.
‘An extra level of kind of torment’: Partners actively excluded from pregnancy loss services and supports.	‘And even this time I know COVID doesn't help but with COVID like its 100 times worse because you have to go alone. And I mean I think they should really consider that you know. I understand with COVID, and you know people going to the hospital and whatever, but I mean I think we really need to reconsider that……I was there alone. Nobody to offer me a word of comfort. Nobody to, I just felt like oh my God this is so wrong. Which it is. I mean it's so bad…And I had been waiting, I was waiting for hours, I must have been waiting close to three hours. And eventually I said to the midwife I said I'm leaving I said I can't wait anymore you know I said this is just too upsetting. I said its actually inhumane to leave a woman like this. And she said no look I'm really sorry. So, then she brought me into the early pregnancy clinic behind a curtain which wasn't much better’. (PW1)	1.1
	‘I felt personally I mean, more on the outside and I think it's partly the reason why I struggle to deal with it because it almost seems like it's not real or it's not happening…disconnected, that's exactly how I've kind of felt at times. Because when I have brought it up to say friends or anyone and even some colleagues that were aware that it happened and have been through similar things they were kind of the same. They couldn't really relate because you know they had gone through it with their partners. So, they were kind of saying Jesus that's an extra level of kind of torment’. (PM5)	1.2
	‘…I suppose it was hard because [Wife] was going through this on her own. You know, she was in the hospital. I couldn't be there with her. I was in the waiting room. You know, I left when she was brought in on a wheelchair and her bent over and crying in pain, you know. And for me just to stay at the doors and let her go through that on her own, like it is, it's very hard you know. You know, and I know it's difficult on [Wife], but I think it was difficult on myself because I was back at home here and I didn't know anything. I didn't know what was happening, was she alright, like she lost so much blood, did she haemorrhage. Like I knew nothing. You know, and it was hard to keep focused at home here with three kids, and try to keep them, they were asking for stuff, and at the same time at the back of your mind you were like well maybe she's lying there haemorrhaging and the doctors are all around her and something's seriously wrong and I'm here, not knowing. Like you know it's scary’. (PM3)	1.3
	‘But for that ultrasound it was [Wife] was in there and she might get the bad news by herself. So that was the toughest one me sitting there and waiting for [Wife] to come back into the car and tell me…Maybe if people could go in for those kind of scans where okay someone might be getting really bad news here. Because just getting the bad news, having to walk out of the hospital and walk up to the car then by yourself that's just really tough’. (PM4)	1.4
	‘COVID has brought an awful slant on it, the way the partners aren't allowed in. So, the grief. The grief some of the women are going through its horrible. You know and I'm hoping that will change soon. And they're not allowed in for the scan and I know the women are really finding that difficult. And I think the partners are. You know they keep saying it’. (M1)	1.5
	‘But then when I went back in the early hours into A&E they did let him come into the cubicle. And he was allowed to stay with me until we went up to the EPU [early pregnancy unit] but he wasn't allowed in for that scan. And then when they said I was staying in they'd also said to me you know he won't be able to see you now, he'll have to drop in a bag. But actually, the nurse who was minding me on the gynae ward because I was in a private room she was like no he can come in, no we have to be human. And so, she did allow him in, and she didn't put a time limit on it either. She was like listen as long as he's got a mask on, and he's washed his hands and sanitised she was like I have no problem’. (PW14)	1.6
	‘…for our first son I was there for every scan, every single appointment. I didn't miss one of them and it was great, and it was fantastic. It was good for me to feel part of it and see things and be there to support [wife]. And this time around I mean I'm in health care myself. I understand we must protect staff. I understand we must reduce footfall. I understand we must protect people and I totally agree with that…… But I think pregnancy loss is just such a sensitive topic that you know there needs to be special arrangements around that. And the very hard thing for [Wife] was that you know you look at [Hospital 3] they allow partners in for scans, partners post‐partum for visits. And in [Hospital 4] it's a blanket no. I know there was a brief reprieve where they allowed people in for the anomaly scan but to know that if you were living in a different part of the country you could have support. I understood the fact that I couldn't be there, and I didn't mind that. But once it turned to pregnancy loss you know dropping her off at the hospital that day for the ERPC [‘evacuation of retained products of conception'] and leaving her on her own to go through that was very difficult…… It's harder for [wife] but it's not a nice position to be in to see your partner being so vulnerable and having to let them to go into that environment vulnerable’. (PM8)	1.7
Grieving in isolation.	‘I always say to people you know it's like that disenfranchised grief that because you know people haven't seen your baby or actually even seen you pregnant or acknowledged that and you don't know where to go with it. And particularly now because of COVID they haven't been meeting their families. They haven't got that support. They haven't got their friends. And the nature of grief and trauma anyway is the fact that your whole world is at a standstill and everything else flies by…So people are at home isolated’. (BM3)	1.8
	‘You're way more isolated because you know I mean you feel very isolated anyway after having a miscarriage because people don't wanna talk about it. Nobody wants to talk about dead babies. Nobody wants to go there. So, you feel isolated anyway and then you're further isolated because you know you can't go out, you can't do the things you'd normally do. I mean you can't even go to a shop for a bit of retail therapy. So definitely you know I'm sitting at home stuck in the four walls most of the days’. (PW1)	1.9
	‘I think the lockdown has made it particularly more difficult, not having that, you know, family and friends support’. (PM8)	1.10
	‘So yeah it was the isolation I suppose made it worse as well. You know which can't be helped either. But it was just even with the girls we couldn't leave them with somebody because of the restrictions and then I was like I don't wanna get somebody in trouble because of the restrictions and left them where they shouldn't be. So, the girls were actually in the house that day [they had a miscarriage]. Now the two‐year‐old doesn't care as long as she's got food. But the older one was wondering what was going on. You know she knew something wasn't right. So, you know whereas I felt like if we'd had that bit of space even to send them off for a day it would have made a difference I think to how I coped with that as well’. (PW14)	1.11

**Table 2:** Theme 2: The perceived dispensability of recurrent miscarriage services and supports.

Subtheme	Illustrative quote	Quote no.
Deprioritisation of recurrent miscarriage services.	‘I suppose as a support midwife I find it really difficult because I know there are those people who need support and aren't getting it. Yeah and particularly, I suppose if we're talking about COVID it has become even more…so I find myself getting really emotional when I talk about that because I think…I was redeployed during the COVID to the scanning department…. but when I came back into it and in to see the impact on people and the impact of no support. You know I mean we are that link for them for support in trying to move forward in planning for future or you know assessing where they're at or assessing their past and plans for the future’. (BM3)	2.1
	‘I think it's the one service that seems to be so quickly movable you know in terms even of just what's happening locally in terms of well it was the pregnancy loss services and others you know were decamped…I think partly because some people can't deal with it and also because for others its seen as you know well there's not much we can do here anyway. There's almost like a fatalistic inevitability to it’. (CP1)	2.2
The loss of supportive spaces.	‘That's why I think it's really difficult. How do you manage that in order for people to get the proper care that they deserve? And especially now in COVID. Actually, here we have because of I suppose trying to manage time and space and people we've amalgamated our ward downstairs with our gynae ward so that is like now pure madness because you have everything on the spot. So, we are writing letters about that. And I'm sure it won't be forever. But I think it just kind of it would magnify that decision‐making process that goes on really. Do you know what I mean? Because you have women who are pregnant going into labour, and then you have this [miscarriage], and then you have people who are dying over here and it's just bananas’. (BM3)	2.3
	‘And here they are coming into a hospital where they have to pass blue and pink balloons in the shop first of all because they all now have to come in the main entrance because of the COVID restrictions to be asked the questions. Whereas at least in the past you could kind of warn them and say look come in the clinic door at the back, at least you don't have to pass the shop if you find that difficult. You're less likely to hear babies crying. You know I might say that to them if I met them on the ward and I'd be apologising for what they're gonna meet when they come in’. (BM7)	2.4
	‘I said “I'm having a miscarriage, I'm gushing in my pants”, like I was really explicit with him…It was really, really, really awful, and I was scared because I didn't know what was happening to me…at one stage, when I was like on the street, like a midwife did come out to me, and she was like are you okay and I was like “I don't fucking know if I'm okay”…I was like “no, I don't feel okay, I'm bleeding, I'm panicking, I've my daughter here, he won't let me into the hospital, I don't know what's wrong”. But she just didn't know what to do. They weren't allowed let us in. They were told like don't let anyone in the building…I was left bleeding on the street, “no you can't come in, no you can't come in”. Like a bouncer getting into a nightclub. You know it's just really bizarre. And then getting in, being isolated like there's something wrong with me but they're treating potential COVID sooner than they're treating my immediate medical need…’ (PW13)	2.5
Care in virtual spaces.	‘I think the girls [Bereavement Midwives] do talk to them on the phone, and they organise their appointments, so they're getting some support, but it's not as good as it would be if they were going to the clinic. They would get more time, and more compassion I suppose, and the consultant talks to them directly’. (M1)	2.6
	‘You know this COVID business, and all, Zoom, yeah it works, but for me there's something that we are going to lose and it's going to be horrendous actually is that contact and being with people and listening to people is a whole body experience. It's totally a whole‐body experience. You walk into the room and its already happening…you can get a sense of that's okay doing this but that's if you have a good relationship I believe. Because I also have to see clients via Zoom and its okay with my older clients that I've had a rapport and I kind of know them. But the ones that are terrified it's really not as because you cannot create that sense of you know the support or get a sense. So that's what I think we are missing actually. And we probably have been missing it in relation to the couple and the dads and their part in it. So my fear is actually that it will get worse’. (BM3)	2.7
	‘Obviously with the COVID thing now we weren't doing face‐to‐face stuff really much at all. That was one thing that was missed I suppose during the COVID crisis…… Initially I suppose we were cancelling appointments and putting them on our waiting lists and organising it, but then we developed the virtual clinics so that made it a little bit easier. And I suppose it lessened the wait time and reduced the anxiety for those patients as well’. (BM5)	2.8
Service improvements shelved.	‘It kind of got shelved during COVID and this is terrible because everything seems to have. There is a huge vacuum arriving out of COVID’. (BM4)	2.9
	‘You know and the public meeting that we had, our next one was going to talk about recurrent miscarriages, so I was dying for that to happen—we had to cancel it over COVID’. (M1)	2.10

### 3.1 Theme 1: Disconnected

Our analysis demonstrates that, during the COVID‐19 pandemic and associated social restrictions, women and men with recurrent miscarriage experienced a sense of ‘disconnect’ in various aspects of their lives. Women's partners were actively excluded from pregnancy loss services and supports, and the absence of informal support networks and the routine of daily life meant that couples were forced to grieve alone. These isolating circumstances appear to have compounded the trauma experienced by women and men with recurrent miscarriage.

#### 3.1.1 ‘An extra level of kind of torment’: Partners actively excluded from pregnancy loss services and supports

Our findings highlight the active exclusion of women's partners from pregnancy loss services and supports during the COVID‐19 pandemic because of the restrictions enforced in maternity settings. Health professionals and people with lived experience of recurrent miscarriage, described how partners were excluded at the point of miscarriage, from recurrent miscarriage clinics, fertility services and from antenatal care in pregnancy after loss and they noted that this heightened the fear and ‘anxiety’ that both women and men experienced. Findings indicate that many women were left ‘vulnerable’ and alone to navigate the physical and emotional aspects of recurrent miscarriage, as well as the associated services (see Quote 1.1; Table 1).

The men in our study expressed grave concern for their partners and significant distress at not being granted access to support them, with many describing waiting unknowing and ‘powerless’ at home or outside of the hospital. Some women outlined how they were forced to break the news of a miscarriage to their partners who were anxiously waiting in the hospital carpark or at the hospital door. Additionally, it was noted that it was challenging for women to absorb the information they were being given by health professionals and to ask questions while distressed and alone, particularly at the point of miscarriage. This sometimes made relaying the details of the loss and/or care plan to their partner difficult, with some men voicing that they were not fully aware of the plans that had been put in place to manage their recurrent miscarriage. Further, some men described how not being present for the physicality of loss or input from health care professionals left them feeling disconnected from reality and with a false sense of hope about the pregnancy. They were left ‘on the outside’, denied access to pregnancy loss services and the opportunity for a connection to be made with supportive care (see Quotes 1.2–1.5; Table 1).

Women and men voiced understanding of the need for restrictions in health care settings, but they struggled to make sense of ‘the blanket no’, particularly given the ‘sensitivity’ and seriousness of recurrent miscarriage. One man felt confused at the presence of a student doctor observing his partner's care while he was excluded from being there to support her, and another questioned the lack of standardisation in restrictions across hospitals meaning that he could have been present to support his partner if they were living in a different county.

Health professionals, as well as women and men in our study, discussed how compassionate exemptions were made to ‘override the system’ in some cases. A Clinical Midwife Specialist noted that, at times, partners were accommodated to support women through medical management and some women and men discussed how they/their partner were allowed to enter as far as the emergency waiting room or to spend some time in a private room (see Quotes 1.6–1.7; Table 1).

#### 3.1.2 Grieving in isolation

Participants indicated that women and men with recurrent miscarriage often experience ‘disenfranchised grief’ because the imperceptible nature of early pregnancy and loss mean that it is not often discussed, and the associated anguish unacknowledged. Health professionals and people with lived experience of recurrent miscarriage described how COVID‐19 social distancing measures further exacerbated feelings of grief and ‘isolation’ as support networks including family, friends and work colleagues ‘were taken away’. Couples were left to grieve at home alone with little to distract them from their thoughts and feelings, in some cases this led to increased personal and relationship strain for women and men. Some parents described how social restrictions prevented them from engaging childcare during lockdown, which complicated their experiences of miscarriage. They felt that they did not have the personal space to adequately process their situations or to connect with and cope with their grief.

Health professionals, and women and men with recurrent miscarriage, noted that unlike women, men do not actively reach out for support but might broach their experience of miscarriage with peers on a social night out. However, this was not possible during the pandemic, thereby closing this outlet for men, leaving them further silenced and isolated. One man noted that although he discussed his situation with friends who had been through miscarriage they could not relate as they had not experienced miscarriage during a time of COVID‐19 lockdown. He shared that this left him feeling further disconnected from reality as if what was happening to him wasn't real. One Clinical Midwife Specialist perceived an increase in the uptake of bereavement support while couples were segregated from their informal support networks (see Quotes 1.8–1.11; Table 1).

### 3.2 Theme 2: The perceived dispensability of recurrent miscarriage services and support

Within this theme, changes to service provision such as service reduction and redeployment, reconfiguration of physical spaces in which care took place, and the indefinite postponing of planned improvements to services were seen to signal a lack of value in recurrent miscarriage services and support. In addition, while virtual clinics facilitated access to services, preference for in‐person care was highlighted.

#### 3.2.1 Deprioritisation of recurrent miscarriage services

Some health care professionals felt that the swift termination or reduction of recurrent miscarriage services and support, and the redeployment of their dedicated staff to other areas, highlighted their perceived dispensability within the health service. Participants described how the pandemic diverted resources away from recurrent miscarriage care (including early pregnancy clinics) leaving a ‘vacuum’ of services and support for those who needed them. They discussed how recurrent miscarriage clinics were suspended in the early stages of the pandemic as well as other supportive services including bereavement support and/or perinatal mental health. Reassurance scans for women pregnant following a loss, valued for helping to ease anxiety with a history of miscarriage, were also cancelled. However, one Sonographer admitted to providing reassurance scans to women with a history of three more miscarriages despite this being prohibited during restrictions because she felt it was unfair to refuse them while in distress. In the later stages of the pandemic where in‐person clinics were in operation, a reduced number of appointments were conducted to facilitate social distancing in waiting areas. These issues resulted in increased waiting lists and prolonged anxiety for those waiting/hoping to attend. Additionally, health professionals noted that phlebotomy services were limited, and blood was not routinely taken due to the conduct of virtual clinics only. Where blood‐testing was provided, increased demands on the analytical laboratories were thought to have caused delays in processing. Health professionals noted that couples were advised to attend their GP to get their bloods done, with one Consultant noting that a lot was being asked of GPs during this time. This overburdening of GPs was felt first‐hand by some of the women in our study in that they highlighted accessibility issues. One woman with recurrent miscarriage was made to feel ‘neurotic’ after being berated for following standard practice by contacting her GP for an appointment to confirm a pregnancy. Another woman hoping to conceive again following a loss, wanted to contact her GP after her questions about miscarriage went unanswered by the hospital but she felt that as GPs were so busy due to COVID‐19 she would be wasting his time and so decided to put the appointment off (see Quotes 2.1–2.2; Table 2).

### 3.3 The loss of supportive spaces

Our findings indicate that it can be traumatic for women with recurrent miscarriages to be in environments focused on maternity care or babies. Health professionals described how they make a conscious effort to shield women from this ordeal if possible. However, Clinical Midwife Specialists felt that efforts to manage COVID‐19 in healthcare settings encroached on those spaces that were designed to protect those with pregnancy loss. Some Clinical Midwife Specialists described how planned routes to recurrent miscarriage clinics that were intended to avoid antenatal clinics, crying babies and pink/blue balloons, were lost as attendees were required to enter through designated entrances to complete COVID‐19 screening questions. Others felt that the amalgamation of wards and the resultant complications in the management of ‘time, and space and people’ was a threat to the provision of quality care to those experiencing miscarriage (see Quotes 2.3–2.4; Table 2).

A woman with recurrent miscarriage described being refused access to a maternity hospital by a security guard while experiencing her first miscarriage when restrictions were first introduced. She recalled being left to bleed on the street with her young daughter in her care and felt that COVID‐19 was prioritised over her ‘immediate medical need’ (see Quote 2.5; Table 2).

#### 3.3.1 Care in virtual spaces

Participants described, with mixed reviews, how virtual clinics were introduced via phone or videocall to facilitate the investigation and follow‐up of recurrent miscarriage, and the taking of medical histories for pregnancy bookings following miscarriage. One Clinical Midwife Specialist felt that virtual clinics worked well because it saved people the difficulty of having to return to the maternity hospital for bereavement support. Women and men awaiting investigation or follow‐up for recurrent miscarriage appeared to be grateful for the opportunity to engage with health professionals if only remotely, although it is worth noting that participants indicated they do not often voice concerns or complaints with the service as they are just glad to have access to any help.

Beyond hospital care, a support group representative explained that, despite technical difficulties and a learning curve on their introduction, virtual meetings would be retained going forward as they were thought to facilitate access to support particularly for those in rural areas or those who did not have a meeting in their vicinity. While virtual clinics were thought by some to be a suitable portal for action, support and reassurance, others perceived in‐person services and supports to be superior with the sharing of physical space thought to be paramount in the delivery of quality and compassionate care (see Quotes 2.6–2.8; Table 2).

#### 3.3.2 Service improvements shelved

Participants highlighted various proposals and initiatives to optimise pregnancy loss services and supports that were ‘shelved’ due to the redistribution of already limited resources to COVID‐19 operations. Those in governance and management roles, as well as nursing and midwifery staff, highlighted that advocacy efforts for improved and dedicated spaces for pregnancy loss were side‐tracked and/or thwarted by a reduction in funding opportunities. Participants noted that money for service improvements was scarce during the pandemic and perceived that this would continue to be the case in its wake.

Some service providers explained that routine training days and/or meetings with educational aspects related to recurrent miscarriage/early pregnancy loss were cancelled. Of this group, a minority noted an eventual move to virtual meetings or the development of prerecorded lectures to be used in the absence of face‐to‐face teaching. Some Clinical Midwife Specialists expressed disappointment at the forced cancellation of public engagement events that were intended to inform service improvements relating to recurrent miscarriage (see Quotes 2.9–2.10; Table 2).

## 4 DISCUSSION

Our analysis illuminates views and experiences of miscarriage and recurrent miscarriage care during the initial phases of the COVID‐19 pandemic. Importantly, it draws on insights from people with lived experience, and those involved in the delivery of services and supports. The impacts were wide‐reaching, with impacts at multiple levels, including individual (people with lived experience and staff), interpersonal, community, organisational and policy. Participants felt ‘Disconnected’—many women navigated miscarriage diagnosis and management and care in subsequent pregnancies alone, while men struggled with not being present to support their partners. Our analysis also underlined the perceived dispensability of miscarriage/recurrent miscarriage services and supports in the prioritisation of national healthcare service; staff were redeployed, development halted and services and supports deprioritised. This research adds to the existing body of knowledge regarding impacts of COVID‐19 on maternity services and care experiences, by providing important insights into the under‐investigated experiences of miscarriage care, from presentation with a miscarriage at emergency departments, early pregnancy scans and care, to specific experiences regarding recurrent miscarriage care.

Miscarriage is associated with increased anxiety, depression, posttraumatic stress disorder and suicide.^29^ International guidelines recommend supportive care provision for women/couples with recurrent miscarriage;^52^ it should be advocated for and recognised in both general and contingency health service planning. Both healthcare staff and women and men, partners and support persons, in our study, were negatively impacted by restrictions in maternity services and the deprioritisation of miscarriage care during the pandemic. Greater commitment from those involved in health service planning and funding is needed to ensure that provisions are made to ensure that service provision—including staffing and staff wellbeing—and care experiences are not compromised during any future pandemics. This includes access to early pregnancy care, emergency department care, recurrent miscarriage care (including clinics and investigations) and care in pregnancies following miscarriage (including early pregnancy scans). Ensuring the presence of a partner/support person is prioritised to protect the mental health of people experiencing pregnancy and miscarriage,^13^ as well as attending specifically to partner's needs, is also needed. Men often assume and/or are assigned the role as the protector and supporter of their partner, often to the neglect of their own psychological needs.^53^, ^54^ Participants in our study spoke about how compassionate exemptions were made by staff to minimise or alleviate the impact of restrictions. A recent study conducted in the United Kingdom and the Netherlands found that many women who have birth during COVID‐19 experienced restrictions, but their experience was mitigated by staff actions. We reinforce calls for the prioritisation and equitable distribution of individualised care in maternity care policy, rather than reliance of the benevolence of care providers.^55^

Some service providers felt that service reduction and redeployment demonstrated a lack of value, and ultimately importance, placed on services. Further to Perinatal Bereavement Care Audits conducted within the 19 maternity hospitals/units across Ireland in 2020, it was recommended that the redeployment of Bereavement Clinical Midwife/Nurse Specialists to COVID‐19‐related roles and other duties interrupting Perinatal Bereavement Service should be discontinued.^56^ Our study also highlights that while virtual clinics facilitated access to services, in‐person care was preferred—similar to the findings of McKinlay et al.^13^ regarding telehealth and the preferences of people experiencing miscarriage and pregnancy complications. Previous reviews have, however, found that virtual visits in maternity care appear to be well‐received by patients and health care providers, for a variety of reasons, including cost savings and convenience, improvements in waiting times and cancellation rates;^6^, ^57^ alongside no significant differences in clinical outcomes.^57^ In their study of perinatal bereavement care during COVID‐19 in Australian maternity settings, Boyle et al.^3^ noted that while telehealth was generally viewed as acceptable, useful and in some instances preferred, enabling access to face‐to‐face support where required and recognising the challenges of telehealth, including limited ability to attend to nonverbal cues and body language, were important. Further research is needed to explore what aspects of miscarriage and recurrent miscarriage care can be provided remotely, for whom and how, to maximise acceptability and effectiveness.

Within the literature, concerns have been expressed that some changes introduced within maternity care during the COVID‐19 pandemic could be retained into the future, for a variety of reasons, including perceived economic savings or self‐interest in minimising in‐person care.^6^ It is important that services continue to deliver care in line with guidelines and standards, and, in Ireland, that service improvements continue following increased attention since the publication of the Miscarriage Misdiagnosis Review in 2011.^58^ The lack of priority afforded to miscarriage and recurrent miscarriage care, and resourcing, was also highlighted more generally within our larger study^36^ and has been highlighted in relation to pregnancy loss more broadly.^59^ This needs to be addressed, particularly, though not exclusively, given international developments in miscarriage care, including the publication of the Lancet Series in 2021,^60^ and work within Scotland to establish a ‘dignified & compassionate miscarriage service by the end of 2023’.^61^

### 4.1 Strengths and limitations

We adopted several procedures to ensure rigour in this study.^62^ We strengthened the credibility of our analysis through our ongoing reflexivity and discussion of codes and preliminary and final themes within the team (and our RE:CURRENT Research Advisory Group); we also provide rich descriptions of our methods, and analysis which is supported by rich and thick descriptions and participant quotes. We provide details of our context on several levels (study, project, COVID‐19, maternity care) to facilitate considerations regarding the transferability of our findings. Although our study was carried out in the Republic of Ireland, many of our findings are reflected in the international literature regarding maternity care more broadly, providing insights into the transferability of our findings. There is a historic under‐investment and prioritisation within pregnancy loss services and support in Ireland, with poor care experiences reported.^63^, ^64^ These are not unique to Ireland; poor care experiences regarding miscarriage/recurrent miscarriage care have been reported elsewhere.^65^, ^66^, ^67^

## 5 CONCLUSIONS

Our analysis provides rich insights into the significant impacts that the COVID‐19 pandemic has had on the way recurrent miscarriage care is provided and experienced within the Republic of Ireland with important implications for early pregnancy, miscarriage and recurrent miscarriage care. While our findings reflect similar experiences across maternity care more generally, and across many countries internationally, the deprioritisation of miscarriage services is of particular concern. Services have undergone significant changes and, while these may be temporary, how services should be delivered in the future requires consideration, particularly given the deficits in care and care experiences highlighted prepandemic. It is important that all stakeholders, especially people with lived experience of miscarriage and recurrent miscarriage, are involved in these discussions regarding how services are structured, organised and delivered now, and that these are also future‐proofed for future pandemics.

## AUTHOR CONTRIBUTIONS

**Conceptualisation**: Keelin O′Donoghue, Rebecca Dennehy, Marita Hennessy. **Methodology**: Rebecca Dennehy, Marita Hennessy, Jennifer Ui Dhubhgain, Con Lucey, Keelin O′Donoghue. **Funding acquisition**: Keelin O′Donoghue. **Investigation**: Rebecca Dennehy, Marita Hennessy. **Data curation**: Rebecca Dennehy, Marita Hennessy. **Formal analysis**: Rebecca Dennehy, Marita Hennessy, Jennifer Ui Dhubhgain, Con Lucey, Keelin O′Donoghue. **Project administration**: Rebecca Dennehy, Marita Hennessy. **Supervision**: Keelin O′Donoghue. **Writing—original draft**: Rebecca Dennehy (results), Marita Hennessy (introduction, methods, discussion). **Writing—review and editing**: Rebecca Dennehy, Marita Hennessy, Jennifer Ui Dhubhgain, Con Lucey, Keelin O′Donoghue. All authors approved the final version of the manuscript.

## CONFLICT OF INTEREST STATEMENT

The authors declare no conflict of interest.

## ETHICS STATEMENT

Ethical approval for the study—‘Exploring stakeholder views on RM services in the Republic of Ireland’—was granted by the Clinical Research Ethics Committee of the Cork Teaching Hospitals (CREC) (Reference number: ECM 4 (ff) 10/03/2020 & ECM 3 (gggg) 09/04/2020). Participants provided written informed consent before study participation. They did not receive any remuneration for taking part. All methods were carried out in accordance with relevant guidelines and regulations.

## Data Availability

While data were pseudoanonymised for the purposes of this study, participants are still potentially identifiable from transcripts due to their particular roles, lived experiences and/or turns of phrase. The authors are not therefore sharing the data from this study; this is in line with the ethical approval granted, including consent processes.
